# Beneficial Effects of Chymase Inhibition on Cardiac Diastolic Function and Remodeling Induced by Chronic Angiotensin II Stimulation

**DOI:** 10.3390/ijms26178236

**Published:** 2025-08-25

**Authors:** Shiguma Taniguchi, Denan Jin, Hirofumi Morihara, Shunichi Yokoe, Kazumasa Moriwaki, Shinji Takai

**Affiliations:** 1 Department of Pharmacology, Osaka Medical and Pharmaceutical University, Takatsuki-City 569-8686, Osaka, Japan; ompu71123001@s.ompu.ac.jp (S.T.); hirofumi.morihara@ompu.ac.jp (H.M.); yokoe@ompu.ac.jp (S.Y.); shinji.takai@ompu.ac.jp (S.T.); 2 Department of Innovative Medicine, Graduate School of Medicine, Osaka Medical and Pharmaceutical University, Takatsuki-City 569-8686, Osaka, Japan

**Keywords:** chymase inhibitor, angiotensin II, infusion, cardiac function, cardiac remodeling

## Abstract

In addition to its role in angiotensin II (Ang II) production, chymase exhibits various functions, including activation of latent transforming growth factor beta 1 (TGF-β1) and pro-matrix metalloproteinases (MMPs). However, the extent to which these Ang II-independent functions contribute to pathological conditions remains unclear. In this study, we investigated the Ang II-independent roles of chymase in cardiac remodeling and dysfunction. Eighteen male Syrian hamsters, aged 6 weeks and weighing 90–110 g, were used. Exogenous Ang II was administered to a hamster model that mirrors the human chymase-dependent Ang II production pathway, via subcutaneous osmotic mini pumps (2 mg/kg/day) for 4 weeks. A chymase-specific inhibitor, TY-51469 (10 mg/kg/day), was given daily starting 1 day after commencement of Ang II infusion. Evaluation showed that while systolic blood pressure increased significantly, only diastolic dysfunction developed over time. Ang II treatment led to elevated cardiac expression of chymase, TGF-β1, and MMP-2, and increased the number of chymase-positive mast cells, resulting in notable cardiac hypertrophy and fibrosis. TY-51469 effectively suppressed these molecular changes and improved both cardiac structure and diastolic dysfunction, despite continued Ang II exposure. These results suggest that chymase promotes cardiac remodeling and dysfunction not only through Ang II generation but also by activating profibrotic and matrix-degrading factors, such as TGF-β1 and MMP-2.

## 1. Introduction

Heart failure (HF) is a complex clinical syndrome associated with significant morbidity, mortality, and healthcare burden [1,2]. The pathophysiology of HF involves structural, functional, neurohormonal, and cellular abnormalities, often evolving over time [3,4]. Structural changes in hearts with HF include cardiac hypertrophy and fibrosis, with the renin–angiotensin system (RAS) reportedly playing a crucial role in such pathological remodeling. A previous study showed that angiotensin II (Ang II) infusion directly induces cardiac hypertrophy and fibrosis in animal models [5]. In the clinical setting, it was reported that RAS activity increases during the development of chronic HF [6], and a significant positive correlation between mortality and plasma Ang II levels was observed in patients with HF [7]. Moreover, the addition of enalapril, an angiotensin-converting enzyme (ACE) inhibitor, to conventional therapy in patients with severe congestive HF reduces mortality and improves symptoms [8,9], indicating that overproduction of Ang II after RAS activation has harmful effects in HF pathology. The mechanisms by which Ang II induces pathological cardiac remodeling have been clarified in recent years. In cardiac tissue, Ang II type 1 (AT1) receptors are expressed on both fibroblasts and cardiomyocytes. Stimulation by Ang II reportedly activates NADPH oxidase and the MAPK pathway via these receptors, leading to oxidative stress-induced myocardial damage and cardiac hypertrophy [10]. Furthermore, Ang II is believed to contribute to cardiac fibrosis by promoting fibroblast proliferation and trans-differentiation into myofibroblasts, either directly or through interaction with transforming growth factor-beta 1 (TGF-β1), resulting in excessive collagen production by these activated cells, and increasing cardiac stiffness and worsening cardiac diastolic function [11].

Among the components of the RAS, Ang II is a key mediator and is classically believed to be generated mainly through an ACE-dependent pathway. However, several decades ago, Urata and colleagues reported the presence of a new Ang II-forming pathway in human cardiac tissue that is independent of ACE. Interestingly, they also found that approximately 80% of Ang II formation is dependent on this new pathway [12,13]. Subsequent extensive studies revealed that the enzyme responsible for this alternative pathway is chymase, which is stored in mast cells, and exhibits its Ang II-producing activity after degranulation under certain conditions in the extracellular matrix environment [14]. Chymase is a serine protease that, like ACE, efficiently converts angiotensin I (Ang I) to Ang II in humans, monkeys, dogs, and hamsters. In contrast, in rats and rabbits, chymase cleaves Ang I between amino acids 4 and 5, resulting in the production of inactive fragments [14,15]. In addition to its ability to form Ang II, chymase has also been found to enzymatically cleave latent TGF-β1 [16], pro-MMP-2, and pro-MMP-9 [17,18], converting them into their active forms. These diverse functions suggest that chymase activation might contribute to pathological cardiac remodeling through both excessive Ang II production and activation of TGF-β1, MMP-2, and MMP-9. However, the extent to which chymase-induced activation of TGF-β1, MMP-2, and MMP-9—independent of Ang II production—contributes to cardiac remodeling and cardiac dysfunction has not yet been fully investigated.

In this study, we established an Ang II infusion model in hamsters, a species known to possess chymase activity similar to that in humans. We then investigated the extent to which long-term Ang II-induced cardiac fibrosis and hypertrophy, as well as cardiac dysfunction, could be suppressed by administration of a chymase specific inhibitor. Since Ang II promotes cardiac hypertrophy and fibrosis not only directly but also through upregulation of the transcription and synthesis of TGF-β1 and MMPs, which also contribute to such cardiac remodeling [19,20], in this Ang II infusion model, chymase inhibitors might attenuate cardiac remodeling—despite elevated systemic Ang II levels—through suppression of TGF-β1, MMP-2, and MMP-9 activation, rather than by reducing Ang II production alone. Thus, this approach might enable clarification of the proportion of cardiac fibrosis and hypertrophy caused by the action of chymase that is not mediated by Ang II production.

## 2. Results

### 2.1. Changes in Body Weight, Heart Weight, and Systolic Blood Pressure Following Chronic Ang II Infusion

**Figure 1:**
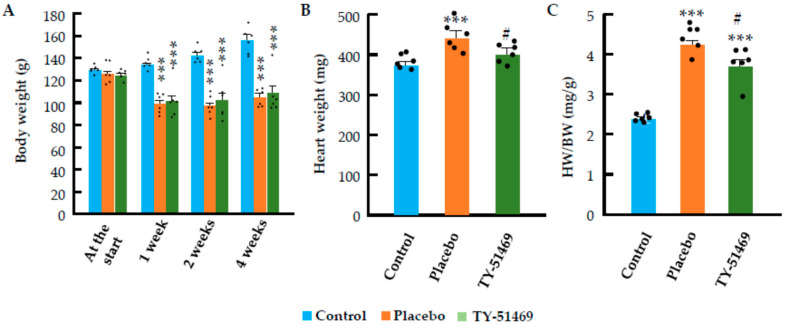
Effects of chronic Ang II infusion on body weight (BW) and heart weight (HW). (**A**): Change in BW in the control group and in Ang II-infused hamsters treated with placebo or TY-51469 during the 4-week observation period. (**B**,**C**): Effects of TY-51469 on absolute HW and HW/BW ratio at 4 weeks after Ang II infusion. *** *p* < 0.001 vs. control; # *p* < 0.05 vs. placebo.

As shown in Figure 1A, there were no significant differences in body weight (BW) among the three groups before implantation of the osmotic mini-pumps. In the placebo-treated group, BW significantly decreased within 1 week after Ang II infusion compared to the control group, but then stabilized and showed a slight increasing trend after 4 weeks. The change in BW over time in the TY-51469-treated group was similar to that in the placebo-treated group.

Figure 1B,C show the absolute heart weight (HW) and HW-to-BW (HW/BW) ratio at 4 weeks after implantation of the osmotic mini-pumps. As illustrated in the bar graphs, both absolute HW and HW/BW ratio were significantly increased in the placebo-treated group compared to the control group, indicating the development of cardiac hypertrophy following chronic Ang II infusion. In contrast, both absolute HW and HW/BW ratio were significantly lower in the TY-51469-treated group than in the placebo-treated group, suggesting that chymase inhibition suppressed the progression of cardiac hypertrophy induced by chronic Ang II stimulation.

**Figure 2:**
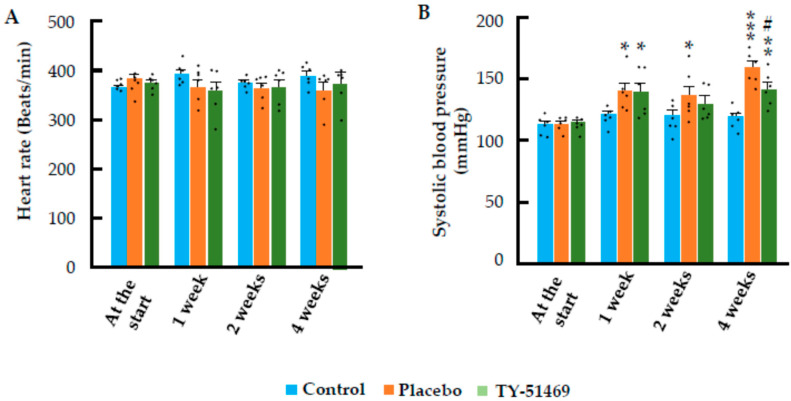
Effects of chronic Ang II infusion on heart rate (HR) and systolic blood pressure (SBP). (**A**,**B**): Changes in HR and SBP in the control group and in Ang II-infused hamsters treated with placebo or TY-51469. * *p* < 0.05, ** *p* < 0.01, *** *p* < 0.001 vs. control; # *p* < 0.05 vs. placebo.

Figure 2A shows the changes in heart rate (HR) over time before and after osmotic mini-pump implantation. Administration of Ang II or TY-51469 had minimal effects on heart rate, and no significant differences were observed among the three groups throughout the 4-week observation period. On the other hand, systolic blood pressure (SBP) in the placebo group increased significantly 1 week after Ang II infusion, and continued to increase further by week 4 (Figure 2B). In the TY-51469-treated group as well, SBP increased significantly at 1 week after Ang II infusion, although the magnitude of the increase was lower than that observed in the placebo group at 4 weeks, indicating that chymase inhibition might slightly attenuate the Ang II-induced increase in SBP.

### 2.2. Beneficial Effects of Cardiac Chymase Inhibition on Cardiac Function Following Chronic Ang II Infusion

**Figure 3:**
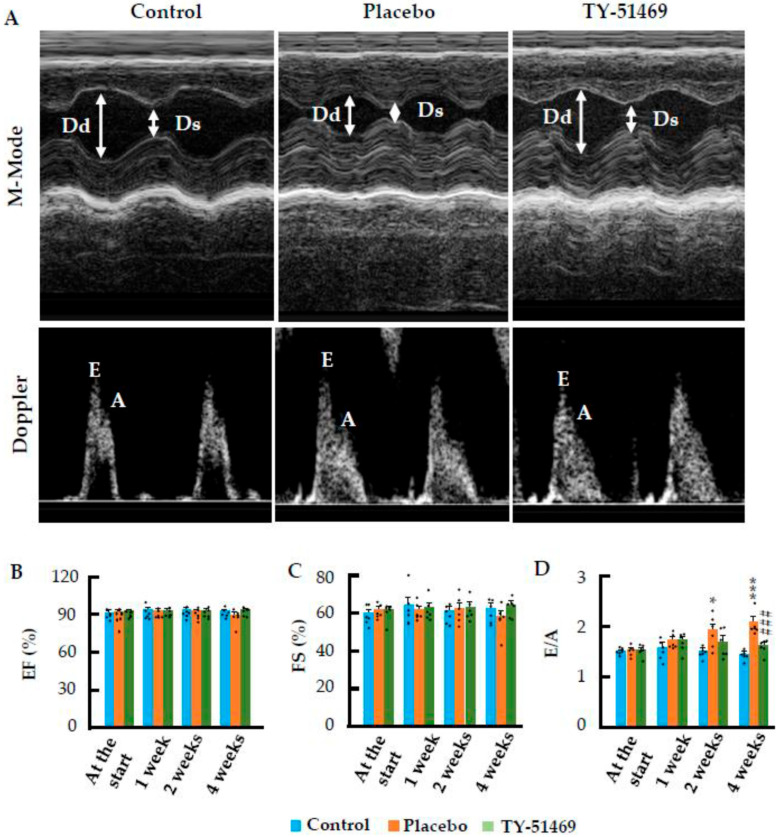
Effects of chronic Ang II infusion on left ventricular ejection fraction (LVEF), fractional shortening (FS), and the ratio of early (E) to late (A) ventricular filling velocities (E/A). (**A**): Representative M-mode echocardiograms and Doppler spectra of mitral inflow in the control group and Ang II-infused hamsters treated with placebo or TY-51469. (**B**–**D**): Changes in LVEF, FS, and the E/A ratio in the control group and in Ang II-infused hamsters treated with placebo or TY-51469. Dd, LV end-diastolic dimension; Ds, LV end-systolic dimension; E, early diastolic inflow velocity; A, late diastolic inflow velocity. * *p* < 0.05, *** *p* < 0.001 vs. control; ### *p* < 0.001 vs. placebo.

Figure 3A presents representative echocardiographic images of control and Ang II-infused hamsters treated with either placebo or TY-51469, taken 4 weeks after osmotic mini-pump implantation. As shown in the bar graphs in Figure 3B,C, indices of systolic function, such as left ventricular ejection fraction (LVEF) and fractional shortening (FS), did not show significant differences among the three experimental groups from 1 to 4 weeks post implantation. These results suggest that chronic Ang II infusion does not markedly affect systolic cardiac function. However, as shown in Figure 3D, the ratio of early (E) to late (A) ventricular filling velocities (E/A), an index of diastolic function, tended to increase from 1 week post Ang II infusion, showing further significant increases at 2 and 4 weeks (*p* < 0.05 and *p* < 0.001 vs. the control group, respectively), indicating that chronic Ang II infusion impairs diastolic cardiac function. In contrast, chronic treatment with the chymase inhibitor, TY-51469, significantly suppressed Ang II-induced elevation of the E/A ratio, suggesting that inhibition of cardiac chymase improves diastolic function during chronic Ang II infusion.

### 2.3. Characteristics of Cardiac Fibrosis Induced by Chronic Ang II Infusion

**Figure 4:**
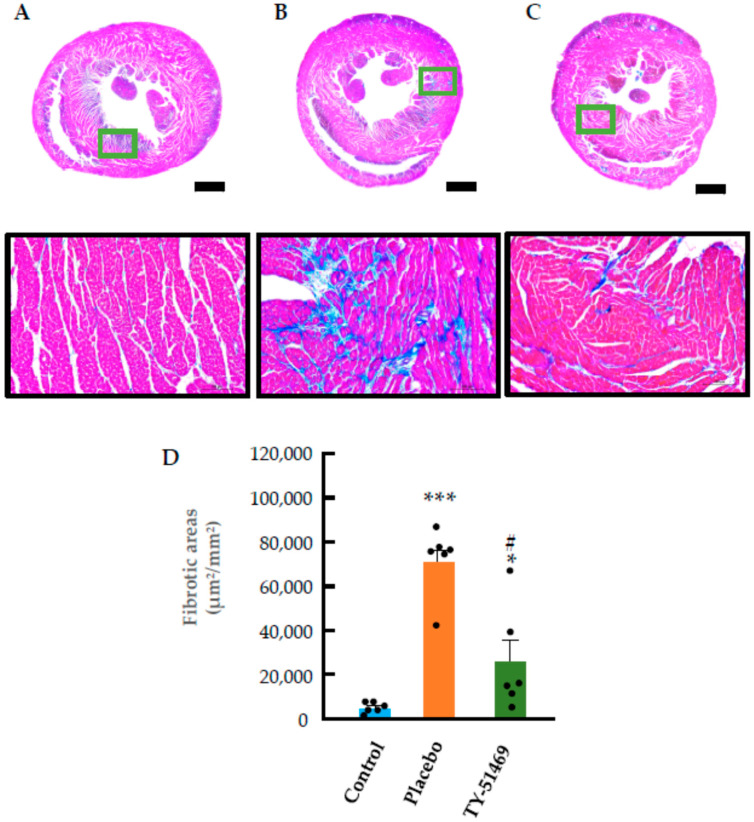
Azan–Mallory staining and quantification of cardiac fibrotic areas. Representative images of Azan–Mallory-stained hearts from control (**A**) and Ang II-infused hamsters treated with placebo (**B**) or TY-51469 (**C**) 4 weeks after osmotic mini-pump implantation. (**D**): Cardiac fibrotic areas in control, placebo, and TY-51469 groups 4 weeks after osmotic mini-pump implantation. * *p* < 0.05, *** *p* < 0.001 vs. control; # *p* < 0.05 vs. placebo. Bar indicates 1000 μm.

Azan–Mallory staining of control hearts revealed normal histology with no significant interstitial cardiac fibrosis, except in the perivascular regions (Figure 4A). In contrast, chronic administration of Ang II in the placebo group resulted in myocardial cell loss and focal replacement fibrosis characterized by collagen deposition, as shown in Figure 4B. This fibrosis was not diffuse but occurred in discrete localized regions. Quantification of fibrotic areas demonstrated that the extent of cardiac fibrosis was significantly greater in the placebo group than in the control group (Figure 4D). However, treatment with TY-51469 markedly reduced these fibrotic areas (Figure 4C,D).

### 2.4. Changes in Cardiac Mast Cells, Chymase-Positive Cells, and Chymase mRNA Expression Following Chronic Ang II Infusion

**Figure 5:**
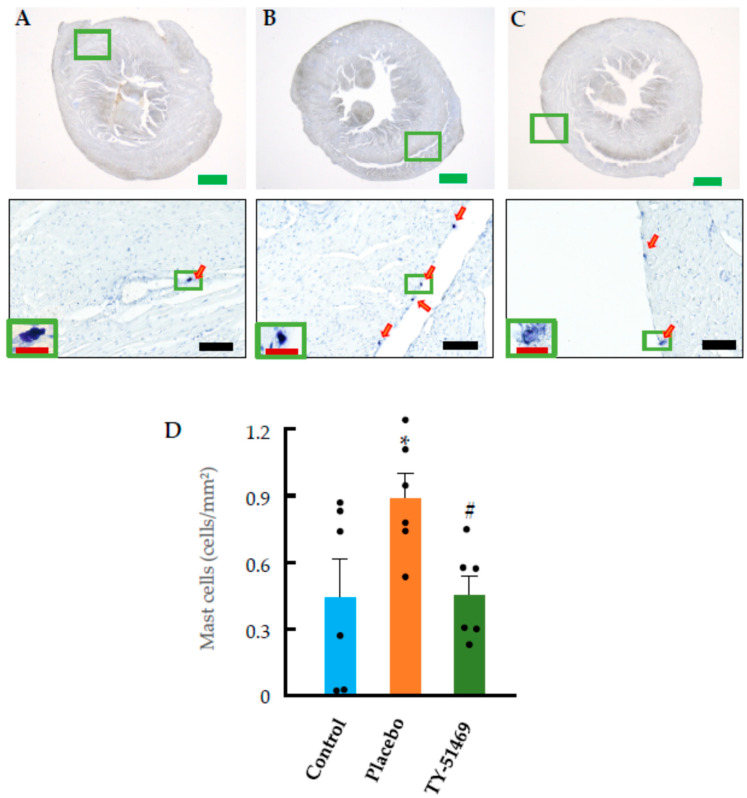
Toluidine blue staining and quantification of mast cells. Representative images of toluidine blue-stained hearts from control (**A**) and Ang II-infused hamsters treated with placebo (**B**) or TY-51469 (**C**) 4 weeks after osmotic mini-pump implantation. (**D**): Quantification of mast cells in control, placebo, and TY-51469 groups 4 weeks after osmotic mini-pump implantation. * *p* < 0.05 vs. control; # *p* < 0.05 vs. placebo. Red arrows indicate mast cells. Green bar, black bar and red bar indicate 1000 μm, 100 μm and 20 μm, respectively.

Figure 5A shows a representative toluidine blue-stained section of a control heart, used for identifying mast cells. Toluidine blue staining is a classical histological method in which mast cells appear metachromatically stained, typically dark purple. As seen in the figure, only a few mast cells were present in the control heart section. However, in the placebo group, the number of mast cells increased significantly 4 weeks after commencement of Ang II infusion compared to the non-infused control group (Figure 5B,D). In contrast, treatment with TY-51469 significantly reduced the number of mast cells compared to the placebo group (Figure 5C,D).

**Figure 6:**
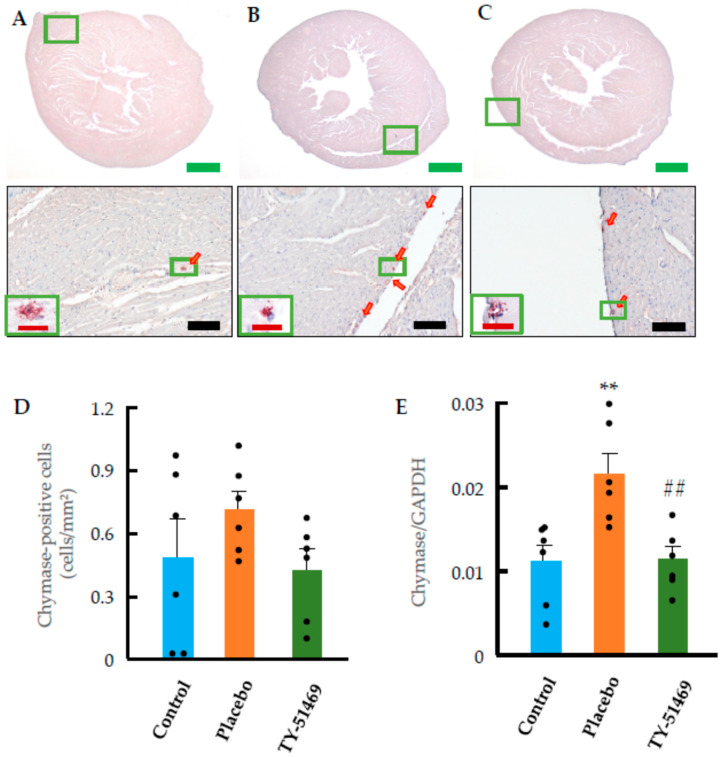
Chymase immunostaining and quantification of chymase-positive cells and chymase mRNA expression. Representative images of chymase-immunostained hearts from control (**A**) and Ang II-infused hamsters treated with placebo (**B**) or TY-51469 (**C**) 4 weeks after osmotic mini-pump implantation. Quantification of chymase positive cells (**D**) and chymase mRNA expression (**E**) in control, placebo, and TY-51469 groups 4 weeks after osmotic mini-pump implantation. ** *p* < 0.01 vs. control; ## *p* < 0.01 vs. placebo. Red arrows indicate chymase-positive cells. Green bar, black bar and red bar indicate 1000 μm, 100 μm and 20 μm, respectively.

Figure 6A shows immunostaining for chymase in a representative control heart. Since chymase is typically stored in mast cells, we prepared serial sections of cardiac tissue to examine the expression pattern of chymase and to identify the cell types expressing it in the hearts of all groups. Consistent with Figure 5A (toluidine blue staining), only a few chymase-positive cells were detected in the control hearts (Figure 6A,D). In contrast, chymase-positive cells tended to increase in the Ang II-infused placebo group (Figure 6B), with a distribution pattern that closely resembled that of the mast cells shown in Figure 5B. Since Figure 5B and Figure 6B represent adjacent serial sections, these results strongly suggest that chymase is primarily expressed in mast cells. Furthermore, the expression of chymase mRNA was also significantly elevated in the placebo group compared to the control group (Figure 6D,E). On the other hand, 4-week treatment with TY-51469 reduced the number of chymase-positive cells and significantly suppressed chymase mRNA expression (Figure 6C–E).

### 2.5. Cardiac Fibrosis Markers and ECM-Degrading Enzyme Expression Following Chronic Ang II Infusion

**Figure 7:**
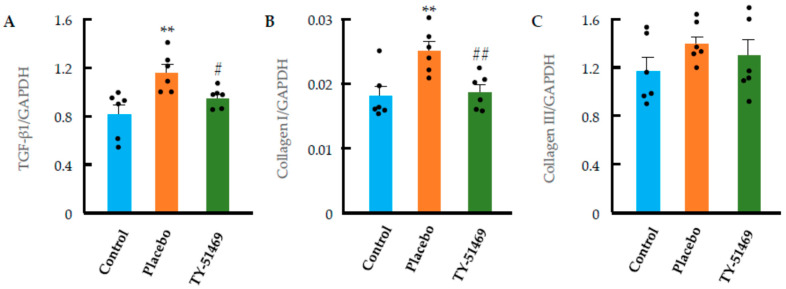
Changes in TGF-β1 (**A**), collagen I (**B**), and collagen III (**C**) mRNA expression in hearts from control and Ang II-infused hamsters treated with placebo or TY-51469 4 weeks after osmotic mini-pump implantation. ** *p* < 0.01 vs. control; # *p* < 0.05; ## *p* < 0.01 vs. placebo.

TGF-β1, collagen I, and collagen III are fibrosis-related factors known to contribute to the development of cardiac fibrosis under pathological conditions. Figure 7A shows the mRNA expression levels of TGF-β1 4 weeks after osmotic mini-pump implantation. As indicated in the bar graph, TGF-β1 expression was significantly elevated in the Ang II-infused placebo group compared to the non-infused control group. However, this increase was significantly suppressed by treatment with TY-51469.

Collagen I and III are key components of the extracellular matrix in fibrotic tissues, and their production is primarily regulated by TGF-β1. As shown in Figure 7B, collagen I mRNA expression was markedly increased in the Ang II-infused placebo group, while this upregulation was significantly attenuated by TY-51469 treatment. Collagen III expression also showed an increasing trend in the placebo group, although the change was not statistically significant. These findings suggest that collagen I might play a more dominant role than collagen III in the development of cardiac fibrosis induced by chronic Ang II infusion. Notably, collagen III expression was not significantly affected by TY-51469 treatment.

**Figure 8:**
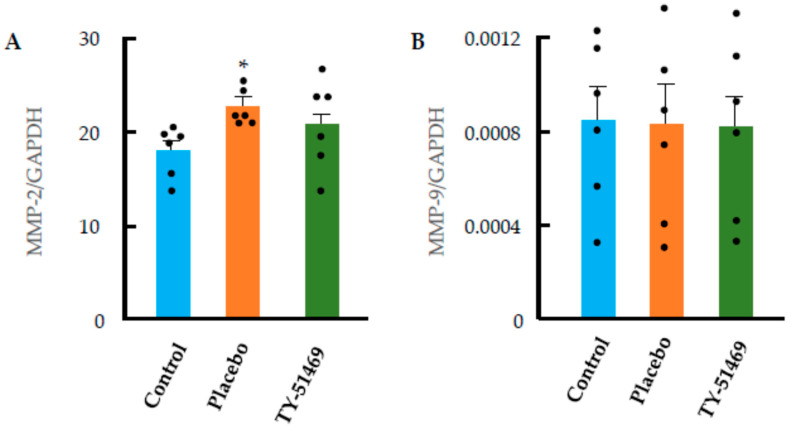
Changes in MMP-2 (**A**) and MMP-9 (**B**) mRNA expression in hearts from control and Ang II-infused hamsters treated with placebo or TY-51469 4 weeks after osmotic mini-pump implantation. * *p* < 0.05 vs. control.

Many studies have shown that MMPs, particularly MMP-2 and MMP-9, are involved in cardiac remodeling processes, such as cardiac hypertrophy and fibrosis. As shown in Figure 8A, cardiac MMP-2 mRNA expression was significantly increased in the chronic Ang II-infused placebo group compared with the control group. On the other hand, the mRNA expression of cardiac MMP-2 tended to decrease following TY-51469 treatment. In contrast, cardiac MMP-9 mRNA expression did not change significantly in the Ang II-infused placebo group, and the expression level in the TY-51469-treated group was not notably different from that in the placebo group (Figure 8B). Taken together, these results suggest that MMP-2 might play a more prominent role than MMP-9 in regulating cardiac remodeling in the chronic Ang II-infused model.

## 3. Discussion

In this study, we investigated the expression profile of chymase and the effects of a chymase-specific inhibitor on cardiac function and remodeling, including hypertrophy and fibrosis, in a hamster model of chronic Ang II infusion. We found that long-term administration of Ang II induced an increase in chymase gene expression and a higher number of chymase-positive mast cells, which was accompanied by significant cardiac hypertrophy and fibrosis, as well as decreased cardiac diastolic function. In contrast, daily administration of the chymase-specific inhibitor, TY-51469, suppressed chymase gene expression, reduced the number of cardiac chymase-positive mast cells, and significantly decreased both the HW/BW ratio and the extent of collagen deposition, as assessed by Azan staining 4 weeks after Ang II infusion. Furthermore, the antifibrotic effect of chymase inhibition was also associated with a significant decrease in the E/A ratio on echocardiography, suggesting that long-term chymase inhibition led to an improvement in cardiac diastolic dysfunction. These findings indicate that chymase activation plays a significant role in the development of cardiac remodeling and the functional decline induced by prolonged Ang II stimulation.

**Figure 9:**
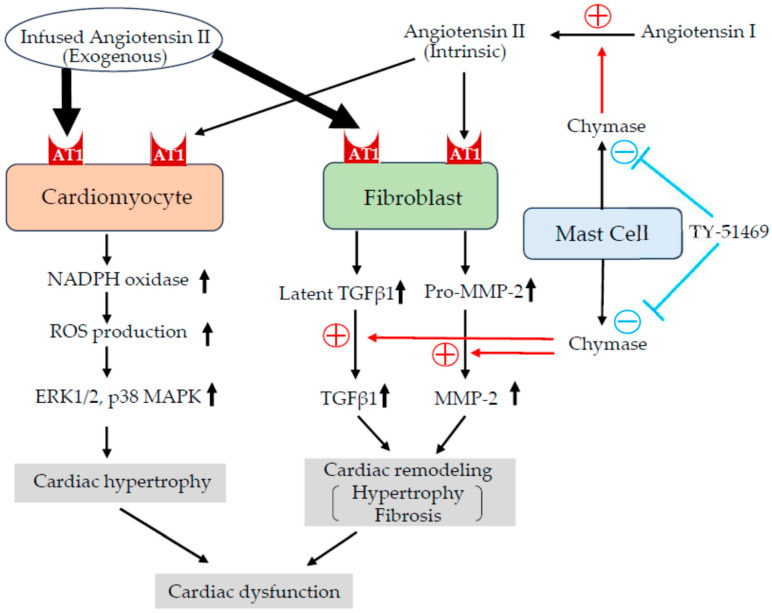
Proposed mechanism of chymase inhibitor (TY-51469) action on angiotensin II (Ang II)-mediated cardiac remodeling. Ang II exerts both direct and indirect effects on cardiomyocytes and cardiac fibroblasts through AT1 receptor activation. In cardiomyocytes, AT1 receptor stimulation activates intracellular signaling pathways (including NADPH oxidase and ROS-MAPK), promoting protein synthesis and cellular hypertrophy. In cardiac fibroblasts, AT1 receptor activation upregulates latent TGF-β1 and ProMMP-2 expressions. Chymase subsequently converts these precursors into active TGF-β1 and MMP-2, driving fibrotic and hypertrophic processes in the heart. The chymase inhibitor TY-51469 blocks the conversion of latent TGF-β1 to active TGF-β1 and of ProMMP-2 to MMP-2, thereby attenuating these remodeling pathways. In this hamster model, where exogenous Ang II was administered in high doses, the inhibitor’s effect on endogenous Ang II production was negligible, with its primary action being through inhibition of the described pathway.

So, by what mechanism does the chymase inhibitor TY-51469 not only improve cardiac function but also suppress cardiac hypertrophy and fibrosis under conditions of high-dose exogenous Ang II administration? Many studies have elucidated the direct and indirect molecular mechanisms of Ang II-induced cardiac hypertrophy and fibrosis (Figure 9). For example, binding of Ang II to the AT1 receptor on cardiomyocytes activates NADPH oxidase, which increases superoxide (ROS) production and activates signaling proteins in cardiomyocytes, such as ERK1/2, p38 MAPK, JNK, and Akt, which increase protein synthesis and gene expression, thereby increasing the size of cardiomyocytes and contributing to the formation of cardiac hypertrophy [21,22]. On the other hand, AT1 receptors are expressed not only in cardiomyocytes but also in various other cell types. Stimulation of these cells by Ang II can lead to increased expression of profibrotic and remodeling-related factors, such as TGF-β1, MMP-2, and MMP-9, potentially promoting cardiomyocyte hypertrophy and interstitial fibrosis indirectly, even in the absence of a direct effect on cardiomyocytes (Figure 9). For example, Ang II has been shown to enhance the gene expression and synthesis of TGF-β1 in cardiac fibroblasts and vascular smooth muscle cells, contributing to organ fibrosis through increased production of extracellular matrix proteins [23]. Additionally, an in vitro study reported that conditioned medium from Ang II-stimulated fibroblasts induced cardiomyocyte hypertrophy, which was attenuated by a neutralizing antibody against TGF-β1 [24]. In an in vivo model, Ang II infusion failed to induce cardiac hypertrophy in TGF-β1 knockout mice, suggesting that TGF-β1 not only contributes to cardiac fibrosis via fibroblast activation but also indirectly promotes cardiomyocyte hypertrophy [25].

In the present study, chronic Ang II administration in hamsters resulted in marked cardiac hypertrophy and fibrosis, accompanied by significant upregulation of TGF-β1 and collagen I expression. On the other hand, treatment with the chymase-specific inhibitor, TY-51469, significantly suppressed TGF-β1 gene expression, which was associated with decreased collagen I expression and marked attenuation of both cardiac hypertrophy and fibrosis. Although cardiac hypertrophy is generally considered proportional to elevations in blood pressure, systolic blood pressure (SBP) in the TY-51469-treated group remained significantly higher than that of the control group (Figure 2B). This finding suggests that the inhibitory effect of chymase inhibition on cardiac hypertrophy occurs largely independently of its impact on blood pressure.

Since chymase is known to activate latent TGF-β1, chymase inhibition would be expected to reduce fibrosis by preventing TGF-β1 activation, even without suppressing TGF-β1 gene expression. However, as shown in Figure 7A, chronic TY-51469 administration led to a significant reduction in TGF-β1 mRNA expression. Although the precise mechanism underlying this unexpected finding remains unclear, it might be partly explained by the autocrine or paracrine characteristics of TGF-β1. For instance, in hereditary gingival fibromatosis, gingival fibroblasts have been shown to rely on a TGF-β1 autocrine loop for their proliferation [26]. Thus, TGF-β1 released from fibroblasts or other cell types might further amplify its own expression and promote fibroblast proliferation and trans-differentiation into myofibroblasts, thereby contributing to the progression of fibrosis. In the present study, TY-51469 treatment reduced fibrotic areas, suggesting that chymase inhibition suppressed both the proliferation and trans-differentiation of fibroblasts. As a result, the TGF-β1 autocrine loop might have been disrupted, which could partially explain the observed downregulation of TGF-β1 expression.

In the present study, Ang II administration led to an upregulation of MMP-2 expression, which was suppressed by treatment with the chymase inhibitor TY-51469 (Figure 8A). Previous studies have reported that Ang II enhances the expression of MMP-2 and MMP-9 in cardiac fibroblasts via activation of transcription factors, such as NF-κB and AP-1 [27], and that activation of these enzymes destroys the morphology of the extracellular matrix, resulting in increased vulnerability of cardiomyocytes, leading to apoptosis and reduced contractile force [28]. Moreover, it has also been reported that targeted deletion of MMP-2 ameliorates myocardial remodeling in mice with chronic pressure overload, indicating that MMP-2 plays a critical role in the development of cardiac hypertrophy [20,29]. The present study, demonstrating that chymase inhibition significantly attenuated both cardiac hypertrophy and fibrosis, suggests that suppression of MMP-2 activation might contribute, at least in part, to the beneficial effects of chymase inhibition on cardiac remodeling.

## 4. Materials and Methods

### 4.1. Animals

Eighteen male Syrian hamsters (SLC, Shizuoka, Japan) aged 6 weeks and weighing 90–110 g were included. All experimental procedures were conducted in accordance with the guidelines of Osaka Medical and Pharmaceutical University for medical experiments, and were approved by the local institutional ethics committee (AM24-052). The hamsters were fed a standard diet, had free access to tap water, and were housed in a temperature-, humidity-, and light-controlled environment.

### 4.2. Osmotic Mini-Pump Implantation and Grouping

Osmotic mini-pumps (MODEL 2004, Alzet, Cupertino, CA, USA) filled with an Ang II solution at a concentration of 5 mg/mL were subcutaneously implanted into 12 hamsters under 2.5% isoflurane inhalation anesthesia. The pumps delivered an Ang II dose of 2.0 mg/kg/day. Additionally, starting 1 day after implantation, six of the hamsters received a daily intraperitoneal injection (i.p.) of the chymase-specific inhibitor, TY-51469 (10 mg/kg/day), until the study endpoint of day 28. The remaining six hamsters were given i.p. injections of physiological saline (0.2 mL/body) and served as the placebo group. As a control group, six hamsters were subcutaneously implanted with the Alzet osmotic mini-pumps filled with physiological saline.

### 4.3. Blood Pressure Measurement and Echocardiographic Study

Before and at 1, 2, and 4 weeks after osmotic mini-pump implantation, arterial SBP was measured in all animals (non-invasive NP-NIBP Monitor, MK-2000ST, Muromachi Kikai, Tokyo, Japan) under 2% isoflurane inhalation with an anesthesia machine (NARCOBIT-E (II), KN-1071, Natsume Seisakusho, Tokyo, Japan). After the blood pressure measurements, echocardiographic studies were performed using an echocardiographic system (Vevo 1100 Imaging System, Transducer: MS250, Fujifilm VisualSonics, Toronto, ON, Canada), as previously described [30].

### 4.4. Preparation of Tissue Samples

At 28 days after implantation of the osmotic mini-pumps, all the hamsters were sacrificed with an overdose of sodium pentobarbital (100 mg/kg, intraperitoneal administration) and their hearts were harvested. Subsequently, a transverse 2 mm thick mid-ventricular slice of the heart was fixed in Carnoy’s fixative and embedded in paraffin for histological analysis. The remaining tissues from the basal and apical regions of the heart were stored at −80 °C for subsequent genetic analysis.

### 4.5. Histological Studies

The embedded paraffin blocks were cut into serial cross-sections of 3 μm thickness using a sliding microtome (LITORATOMU, REM-710, Yamato Koki Kogyo Ltd., Asagiri, Saitama, Japan). To assess areas of cardiac fibrosis, the first section from each paraffin block was stained with Azan–Mallory stain. Three areas per section were randomly selected at 200× magnification using a computerized morphometry system (NIS-Elements Documentation, v.3.07, Nikon, Tokyo, Japan). The average fibrotic area was then quantified using an image analysis software (WinROOF2021, MITANI corporation, Tokyo, Japan). To evaluate mast cell distribution, the second section from each paraffin block was stained with toluidine blue. Briefly, after deparaffinization with Remosol (Wako Pure Chemicals, Osaka, Japan), sections were immersed in 0.5% toluidine blue solution (pH 4.8) for approximately 15 min, fractionated with 0.5% glacial acetic acid solution, and mounted after drying. To determine chymase distribution, immunohistochemical staining was performed on the third serial section using an anti-hamster antibody (raised in rabbit by immunization with SPYVPWINIVIKASS, which is the sequence of the C-terminal amino acid residues from positions 212 to 226 of hamster chymase, kindly given to us by Otsuka Pharmaceutical, Tokushima, Japan), following a previously described protocol [31]. Briefly, to suppress endogenous peroxidase activity and nonspecific binding, deparaffinized sections were sequentially incubated with 3% hydrogen peroxide and a protein-blocking solution for 5 min each at room temperature. The sections were then incubated for 1 h at room temperature with the diluted primary antibody (1:100), followed by detection using a labeled streptavidin-biotin peroxidase kit (Dako LSAB kit, Carpinteria, CA, USA) and 3-amino-9-ethylcarbazole (AEC) for color development. Sections were lightly counterstained with hematoxylin and mounted with cover glasses. Following staining, mast cell and chymase-positive cell counts were evaluated under a computerized morphometry system, and the number of cells per unit area was compared between experimental groups.

### 4.6. Real-Time Polymerase Chain Reaction (RT-PCR)

RT-PCR was used to examine the expression of chymase, TGF-β1, MMP-2, MMP-9, collagen I, and collagen III in cardiac tissues, using previously described methods [30]. Briefly, total cardia RNA was extracted using Trizol reagent (Life Technologies, Rockville, MD, USA) and dissolved in RNase-free water (Takara Bio Inc., Otsu, Japan). One microgram of total RNA was transcribed into complementary DNA (cDNA) using Superscript VIRO (Invitrogen, Carlsbad, CA, USA). mRNA levels were then quantified by RT-PCR using a Stratagene Mx3000P system (Agilent Technologies, San Francisco, CA, USA) and TaqMan fluorogenic probes. RT-PCR primers and probes for chymase, TGF-β1, MMP-9, collagen, and GAPDH were designed by Roche Diagnostics (Tokyo, Japan). The primer sequences were as follows:

Chymase: Left: ggacaaaggggcctgtaaat; Right: gtatgctgatctgaccttcgtg.
TGF-β1: Left: gctaccatgccaacttctgc; Right: ccaggaccttgctgtactgtg.
MMP-2: Left: gggagctcaggccagaat; Right: ttggcggacagtgacactac.
MMP-9: Left: cttcgacgacgacgagttg; Right: ttgcgtttccaaagtaagtgg.
Collagen I: Left: tggaccttgttcacctctctc; Right: ccctgctggcaaagatgta.
Collagen III: Left: caccacgctcgccattat; Right: tgggcctcaaggtattcaag.
GAPDH: Left: agcttgtcatcaacgggaag; Right: gcatcaccccatttgatgtt.
The probe sequences were as follows:
Chymase: tgggcagc.
TGF-β1: gagcctgg.
MMP-2: ggccacca.
MMP-9: ctgggcaa.
Collagen I: cagcagga.
Collagen III: ctggctcc.
GAPDH: catcaca.

These mRNA levels were normalized to those of glyceraldehyde-3-phosphate dehydrogenase (GAPDH).

### 4.7. Statistical Analysis

All numerical data are expressed as the mean ± standard error of the mean (SEM). Statistical differences among multiple groups were assessed using one-way analysis of variance (ANOVA) followed by Fisher’s post hoc test (Statcel software). A *p* value of < 0.05 was considered statistically significant. Correlations were analyzed using Pearson’s correlation coefficient.

## 5. Conclusions

Our study demonstrated that chronic administration of Ang II significantly increased the expression of cardiac chymase, chymase-containing mast cells, TGF-β1, and MMP-2, all of which are associated with the development of cardiac hypertrophy, fibrosis, and diastolic dysfunction. Conversely, treatment with a chymase-specific inhibitor not only suppressed the expression of chymase, TGF-β1, and MMP-2, but also effectively attenuated cardiac remodeling and diastolic dysfunction, despite continued stimulation with exogenous Ang II. These findings suggest that chymase contributes to cardiac remodeling and diastolic dysfunction through mechanisms beyond Ang II generation—specifically, through the activation of latent TGF-β1 and pro-MMP-2—highlighting its independent and critical role in the pathogenesis of cardiac fibrosis and hypertrophy. Furthermore, unlike chymase inhibitors, ACE inhibitors and ARBs currently in clinical use do not inhibit the activation of TGF-β and MMP-2, suggesting that chymase could serve as a novel therapeutic target for cardiac remodeling in conditions such as heart failure.

## Data Availability

The data presented in this study are available on request from the corresponding author.
